# Robots in Eldercare: How Does a Real-World Interaction with the Machine Influence the Perceptions of Older People?

**DOI:** 10.3390/s22051717

**Published:** 2022-02-22

**Authors:** Slawomir Tobis, Joanna Piasek, Miroslawa Cylkowska-Nowak, Aleksandra Suwalska

**Affiliations:** 1 Department of Occupational Therapy, Poznan University of Medical Sciences, 60-781 Poznan, Poland; mcylkowska-nowak@ump.edu.pl; 2 Institute of Robotics and Machine Intelligence, Poznan University of Technology, 60-965 Poznan, Poland; joanna.piasek@put.poznan.pl; 3 Department of Mental Health, Chair of Psychiatry, Poznan University of Medical Sciences, 60-572 Poznan, Poland; asuwalska@ump.edu.pl

**Keywords:** social robot, elderly, care, human-machine interaction, acceptance

## Abstract

(1) Background: Using autonomous social robots in selected areas of care for community-dwelling older adults is one of the promising approaches to address the problem of the widening care gap. We posed the question of whether a possibility to interact with the technology to be used had an impact on the scores given by the respondents in various domains of needs and requirements for social robots to be deployed in care for older individuals. (2) Methods: During the study, the opinions of older people (65+; n = 113; with no severe cognitive impairment) living in six social care institutions about a robot in care for older people were collected twice using the Users’ Needs, Requirements and Abilities Questionnaire (UNRAQ): after seeing a photo of the robot only and after a 90–150 min interaction with the TIAGo robot. (3) Results: Mean total scores for both assistive and social functions were higher after the interaction (*p* < 0.05). A positive correlation was found between opinion changes in social and assistive functions (r = 0.4842; *p* = 0.0000). (4) Conclusions: Preimplementation studies and assessments should include the possibility to interact with the robot to provide its future users with a clear idea of the technology and facilitate necessary customisations of the machine.

## 1. Introduction

The ageing of the human populations across the globe affects many fields, among others, also the care sector. As societies age, the demand for assistance in daily activities increases, both globally and from an individual perspective. Another consequence of the ageing of societies is the increasing shortage of caregivers. This so called care gap is projected to grow over time [1]; innovative and efficient solutions for the care of older persons are, thus, urgently needed. Using autonomous social robots in at least selected areas of care for community-dwelling older adults is one of the promising research directions in this field. Such robots can provide support by improving well being and preventing functional decline [2]. However, commercially available products are still in relatively early stages of development and do not fully provide the required solutions [3]. Current assistive service robots are commonly preprogrammed to offer a limited range of services and face difficulties adapting to the changing needs of older individuals [4].

Gathering personal information, making use of them in care, while respecting older people’s choices and employing their past life experience as well as subjective perceptions are vital issues of person centred care [5]. Thus, while developing social assistive robots, researchers and designers should primarily take the point of view of older subjects into account [6]. Besides, it is long established that—to be successful in care for older adults—the robots must be accepted by them [7], which is the more important as robots not only are able to assist their users in the activities of daily living (that is, act as useful devices) but are also likely to influence the social environment of their homes [8] (for example, by providing companionship or stimulating social contacts).

The contemporary paradigm of ageing in place, which includes necessary provisions for independent functioning along the ageing trajectory, is welcomed by the vast majority of older adults who prefer not to move to a care institution for as long as possible [9]. The prospect of being able, thanks to robotic support, to retain ageing persons in their homes (instead of their institutionalisation) fits very well in this concept. Many studies dealing with older people’s acceptance of supportive technologies have been published [10,11]. However, publications that assess the needs and requirements of older adults related to the use of social robots in care have been scarce to date, and so are validated tools for its assessment. Recently, we presented such a tool (the Users’ Needs, Requirements and Abilities Questionnaire—UNRAQ) alongside its psychometric properties, which can be used to collect data about the use of a social robot in care for older individuals from their perspective [12].

The majority of technology acceptance studies published so far performed their assessments without a hands on interaction with the devices in question, which might, to some degree, affect the views of the participants. Henceforth, we posed the question of whether a possibility to interact with the technology to be used had an impact on the scores given by the respondents in various domains of needs and requirements for social robots to be deployed in care for older adults.

## 2. Materials and Methods

During the study, the opinions of older individuals about a robot in care for older people were collected twice using the UNRAQ. The first assessment was carried out before the subjects were exposed to the robot, and the second one was carried out after a 90–150 min interaction with the TIAGo robot (PAL Robotics, Barcelona, Spain).

The project was approved by the Bioethics Committee of Poznan University of Medical Sciences, Poland (Protocol No. 711/18). The participants gave their consent for participation after receiving a full explanation of the nature of the study.

### 2.1. Participants

The studied subjects were conveniently available older individuals (n = 113) living in long term care (LTC) institutions, which are part of the social care sector in the Wielkopolska (Greater Poland) region of Poland. Staying in such a setting is indicated when needs related to everyday functioning are difficult or impossible to meet in the community, possibly also for cost reasons. Therefore, among the inhabitants, there are persons with different physical and cognitive abilities.

Participants from six LTC institutions took part in the study. The inclusion criteria were: age ≥ 65 years and obtaining at least 15 points in the Mini-Mental State Examination (MMSE) cognitive assessment test [13], which is considered necessary to be able to understand the questions and provide adequate answers [14,15]. Obtained MMSE scores were adjusted for age and education [16]. Barthel Index (BI) was used for the assessment of independence in basic activities of daily living [17].

### 2.2. Procedure

In each institution, the basics of the project were presented during an initial meeting. Persons interested in participating were invited to the next meeting, during which each person underwent a cognitive assessment with the MMSE test. For ethical reasons, all individuals willing to participate were assessed; however, we analysed the results only for subjects who obtained 15 points or more in the MMSE score.

Subsequently, the participants expressed their needs and requirements versus a social robot to be used in care for older people using the UNRAQ questionnaire. The UNRAQ has been previously presented, and its good psychometric properties demonstrated [12].

The UNRAQ starts with the characteristics of the participant (such as age, sex, level of education, being a caregiver of an older person, familiarity with technology and ability to operate a computer). The second part is divided into four areas:

- Interaction with the robot and technical issues (10 statements),
- Assistive role of the robot (13 statements),
- Social aspects of using the robot (6 statements),
- Ethical issues (5 statements).

Each area consists of several statements. The participants are expected to express their level of agreement (or disagreement) with each of these statements based on a 5-point Likert scale (1—I strongly disagree, 2—I partially disagree, 3—I neither agree nor disagree, 4—I partially agree, 5—I strongly agree), where scores 4–5 are considered positive. The structure of the questionnaire ensures that the results can be expressed as means and standard deviations (SD). Each statement presents the participant with the possibility to comment in a free form in an extra box provided next to it. The final part of the UNRAQ is the Creativity Box, where any comments, ideas, suggestions, or observations can be put down by the participant that are not reflected in the statements of the questionnaire. The UNRAQ thus combines items of both quantitative and qualitative methodologies.

**Figure 1:**
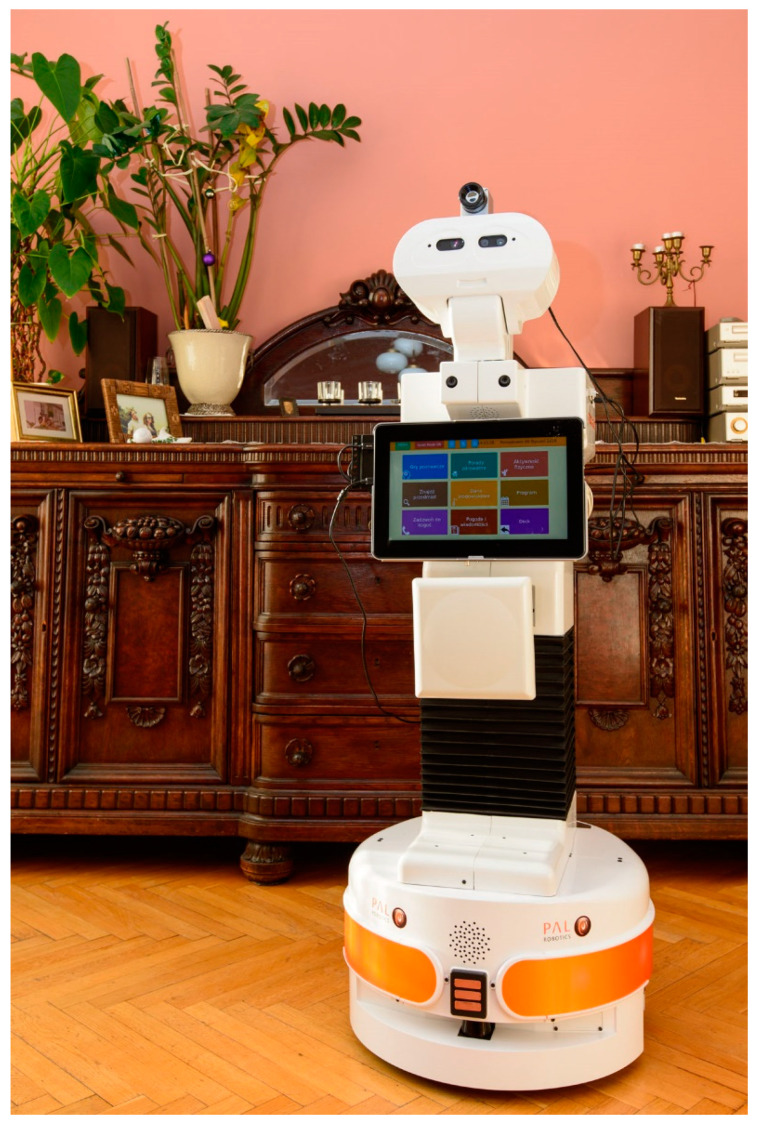
The TIAGo robot.

The next step was the presentation of a humanoid social robot (Figure 1), lasting from 90 to 150 min. The session’s duration depended on the number of participants and their interest in dealing with the robot. The sessions included 11–23 participants and lasted until all interested subjects had sufficient opportunity to interact with the robot. Eventually, the participants completed the UNRAQ once more, following their experience with the robot.

During the presentations, we used a customised version of the TIAGo robot, equipped with a range of sensors (an RGB-D camera with depth recognition capabilities, a thermal camera, an RFID antenna for locating lost objects, a laser scanner, environment sensors, radar distance sensors), a microphone, a loudspeaker and a touch tablet for communication with the user. The robot was wirelessly networked with a remote computer (AIS—ambient intelligence system, connected via the Internet to a cloud based Networked Care Platform). The robot was able to navigate semiautonomously (after creating a precise environment map) or follow the user. Among the options available during the interaction, there were cognitive games, reminders, safety measures (e.g., locking status of doors or the refrigerator), physical exercises, dietary recommendations, video connectivity, provision of news and weather, as well as a readout of environmental values (temperature, humidity, air pressure, air quality etc.).

### 2.3. Statistical Analysis

Statistical analysis was performed with the STATISTICA 13 software (TIBCO Software, Poland). Variables were expressed as percentages for frequencies and means ± standard deviation (SD) and medians. The normality of data distribution was examined with the Shapiro–Wilk’s test.

For the UNRAQ questionnaire, mean scores of social and assistive functions were additionally calculated for each participant. The calculation scheme for social functions (to which six statements are assigned) is presented below:

C = (C1 + C2 + C3 + C4 + C5 + C6)/6

where C is the calculated value for opinions on the social functions of the robot for a given person and C1…C6 respective numerical scores for the individual functions on the Likert scale. The same was performed for the assistive functions.

Comparison between two paired groups of data was made with the Wilcoxon test and differences in the distribution of quality variables between two groups, with the χ^2^ test with Yates correction due to small sample size. The Spearman coefficient was used as a measure of correlation in data. Furthermore, *p* < 0.05 was considered statistically significant.

## 3. Results

### 3.1. Characteristics of the Studied Group

The mean age of participants was 76.6±8.7 years. The oldest studied subject was 94 years old, and 48 persons were at least 80. Most of the respondents were women (n = 64; 56.6%) and unmarried people (widowhood 56.6%, singles 34.5%). Forty-seven people (41.6%) had their education below secondary (28 had primary education—24.8% and 19 vocational—16.8%). Among the rest, only 15 subjects had higher education (13.2%).

Among the surveyed, 49 people (43.8%) stated that technology was, for them, easy to use. As for the health status, 37 people declared it below average, and 42 people (37.2%) assessed their fitness above average.

The mean MMSE score was 23.3 ± 4.1 points; 50 subjects had scores below 24 points; among them, 22 had scores below 20. The mean BI value was 80.0 ± 20.7 points; 56 participants had a BI score of 85 points or more; none of the studied individuals was entirely dependent for the basic activities of daily living.

### 3.2. Opinions of the Participants about the Robot after Viewing Its Photograph Only

The UNRAQ results are presented in Table 1. The respondents rated the robot the highest as *a useful device* (statement A3, 4.3 ± 1.2) and the lowest as *a companion* (A1, 3.9 ± 1.4; *p* < 0.05). Based on the UNRAQ results, the acceptance of the robot by all participants was good as a whole. Only in area A (interaction with the robot and technical issues), we observed mean scores below 3.0; the respondents claimed that older persons were not prepared to interact with the robot and not very good at handling it (A4, 1.8 ± 1.0 and A5, 2.6 ± 1.3, respectively). Statement A6: *The elderly want to increase their knowledge about the robots to be able to operate them* gained much better scores (3.6 ± 1.4, *p* < 0.001 vs. two previously mentioned role statements); still, this value was also relatively low.

The worst rated statements in area B (assistive role of the robot) were related to nutrition (B2, B7 and B10), and in area C (social aspects)—statement C1: *The robot could decrease the sense of loneliness and improve the mood of the elderly person* (the only function for which the average score was below 4.0). Additionally, mean scores for assistive functions were higher than for social ones (B: 4.5 ± 0.3 vs. C: 4.2 ± 0.2; *p* < 0.05).

In area D (ethical issues), all statements obtained high scores.

### 3.3. Opinions of the Participants after Interaction with the Robot

After the presentation of the robot and engaging in interaction with it, none of the assessed elements was scored by the participants lower than after viewing the photos only (Table 1).

**Figure 2:**
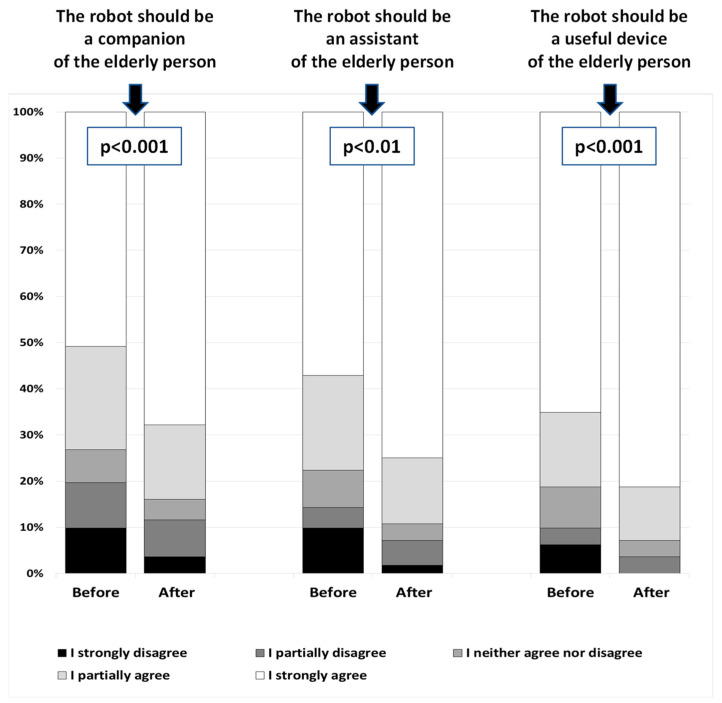
The robot’s roles: scores before and after interaction with the robot.

In area A, all robot roles were rated significantly higher after the interaction (*companion*—*p* < 0.001; *assistant*—*p* < 0.01, *useful device*—*p* < 0.001—Figure 2), although, after the presentation, the *companion* role was still scored lower than that of *a useful device* (4.4 ± 1.1 vs. 4.7 ± 0.7; *p* < 0.05).

The statements A4—The elderly are prepared to interact with a robot—and A8—The robot should be customisable (adjusted to individual user preferences and needs)—were also scored higher after interaction with the robot (*p* < 0.01 and *p* < 0.001, respectively). 

**Figure 3:**
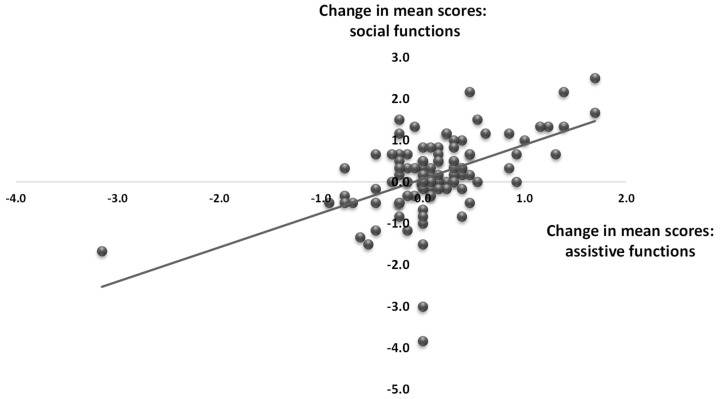
Correlation between opinion changes (before and after interaction) for social and assistive functions.

Within the assistive and social functions in the UNRAQ (areas B and C), only the score of statement C1—*The robot could decrease the sense of loneliness and improve the mood of the elderly person*—increased significantly (*p* < 0.001). However, the mean total scores for both assistive and social functions were also higher after the interaction (area B: 4.5 ± 0.3 vs. 4.6 ± 0.2—*p* < 0.05; area C: 4.2 ± 0.2 and 4.3 ± 0.2—*p* < 0.05). Moreover, a positive correlation was found between opinion changes in social and assistive functions (r = 0.4842; *p* = 0.0000), Figure 3. This means that greater improvement in the score of assistive functions was associated with greater improvement in social functions.

In area D, a higher score after interaction with the robot was obtained for statement D3—*It is acceptable that the robot informs a family member or caregiver about the older person’s behaviour/health problems* (*p* < 0.01).

### 3.4. Investigation of the Determinants of Participants’ Opinion Changes

The analysis of participants whose opinions—in terms of individual statements—worsened compared to the others (Table S1—Supplementary Files) showed that the evaluated parameters had only a slight modifying effect on the change of opinion about the robot after interacting with it. Specifically:

1. age was only relevant for the change of opinion on statement B2—*The robot should help the elderly to preserve their memory function,* e.g., *by playing memory games with them*—opinions of participants from the older group (80 years and older) on this subject were more likely to deteriorate after interaction with the robot in this area (18.8% vs. 6.2%; *p* < 0.05);
2. responses were gender—relevant only for statement D5—*It is acceptable that the robot will have much information about the user* (*social*, *medical*, *others*)—men more often changed their opinion for the worse after contact with the robot compared to women (22.5% vs. 7.8%; *p* < 0.05);
3. ease of use of technological devices was relevant only for statement B4—*The robot should provide advice about a healthy diet*—in people declaring ease of use of technology, a less frequent worsening after interaction with the robot was observed (8.2% vs. 22.2%; *p* < 0.05).

Education, self-perception of fitness and health had no influence on the change of opinion after interaction with the robot. Table S2 (Supplementary Files) presents the UNRAQ results in relation to the scores of MMSE and BI.

## 4. Discussion

Numerous studies have been published to date that assess the opinions of older people on the use of robots in care [10,11]. The vast majority used photographs or video clips to introduce the concept of the robot, and only a few of them studied the prospect of introducing a social robot into the lives of older adults as a caregiver. Our study also started with presenting the participants with a photograph of the TIAGo humanoid robot. Afterwards, we used the UNRAQ questionnaire (which has proven to have good psychometric properties in a large group consisting of various subjects [12]). Based on its results, we observed a good overall acceptance of the idea of using a social robot in care for older people. The acceptance scores are in line with our previous findings [12], those from other studies [18,19,20,21,22,23], and a validated conceptual model, based on the theory of planned behaviour (according to this theory, intention is a central trigger for any behaviour, determined by three factors: attitude towards the behaviour, subjective norm, and perceived behavioural control) [24]. Despite a high general acceptance, older subjects preferred the robot to play a role of a useful device rather than that of a companion. Similarly, Frennert et al. observed that it was difficult for older adults to imagine a robot as a friend [25].

In our current study, after the TIAGo robot had been presented and the participants had had the opportunity to interact with it, all discussed robot’s roles were rated positively, including the role as a companion. In this context, it should be stressed that we particularly observed an improvement in the potential lowering of the sense of loneliness of an older person by the presence of the robot. This role is the more important, as loneliness and social isolation are treated as difficult to address geriatric giants, which affect the functioning of older people in many areas [26,27,28]. It may also mean that the participants were able to imagine the role of the machine in their life and envision the most important benefits of the presence of the robot. Another indication of the improvement of robot’s acceptance after the interaction is the score of statement D5: *It is acceptable that the robot informs a family member or caregiver about the older person’s behaviour/health problems*, which additionally signals a high degree of confidence in the machine’s ability to collect relevant observational data and present it to the person taking care of the robot’s user. On the contrary, in a study on older adults with mild cognitive impairment and cognitively intact healthy ones, Wu et al. showed that direct experience with the robot did not change the way the participants rated robots in their acceptance questionnaire [29]. This observation may have been due to low intention to use a social robot in the studied subjects.

Our results showed that the contact of participants with the robot did not have a statistically significant effect on how the individual functions were rated but influenced the total scores, both for assistive and social functions. In an earlier study, Bedaf et al. observed that older adults were postinteraction positive about the realistic robot use scenario they took part in, even more so than other stakeholders [30]. The scenario involved a robot operating in a smart environment, performing defined assistive tasks. In addition, in an international qualitative study, D’Onofrio et al. stated that the postexposure perception of the usefulness of a robot was positive, even by older individuals who were not familiar with new technologies [31]. In our study, the improvement in scores for both assistive and social functions indicates that older people are better able to envision the potential role of a humanoid social robot in their lives after contact with a real robot. Both scores changed proportionally, but the increase was higher for the assistive functions. This seems to demonstrate that it is still difficult to imagine social support delivered by a machine. One might speculate that the state of technology is not yet perceived as mature enough for this purpose.

Beer et al. observed an improvement in perceived ease of use after a 2.5-hour long exposure to a robot [32]. Chen et al. demonstrated an improvement in perceived ease of use of the robot in long term care residents with dementia after a 32-week interaction with the robot [33]. Likewise, in our study, the improved score for the statement on the preparedness of older adults to interact with the robot demonstrates, on the one hand, that their reservations versus the robot are at least partially due to not being familiar with the technology in question and, possibly, to doubts resulting from “the unknown”. On the other hand, the improvement in the score of the statement related to the need for customisation of the robot to better suit the needs and preferences of its user shows that the participants may find it easier to imagine the role of the robot in their life after actually interacting with the machine, hence also their better defined attitude to the features and functions the robot should have.

Our study has some limitations, among them the one time exposure of the participants to the robot. Therefore, a certain degree of novelty effect may be present [6,34,35]. We included participants with relatively good cognitive functioning, whereas care for those with MMSE scores lower than 15 points is particularly challenging; this group requires a dedicated methodological approach [36,37]. It is, however, imperative to underline the strong points of the study. Its strengths are a comprehensive assessment using a validated tool (UNRAQ) and the inclusion of a substantial number of “older old,” that is, subjects over 80 years of age. Importantly, in our study, a second assessment was performed after a sufficiently timed interaction with the robot. The second score is thus close to real world circumstances in human-robot interaction.

## 5. Conclusions

Older people expressed a good overall acceptance for the use of a social robot in care for their social group. Their contact with the robot had a positive effect on the scores of all assessed robot’s roles, though it still appears difficult to conceive full scale social support delivered by a machine. Through the interaction with the robot, older subjects gained a more detailed picture of its capabilities and were able to relate to the functions and features a potential care robot should have. Preimplementation studies and assessments should, thus, include the possibility to interact with the robot to better prepare its future users for deployment and provide for necessary customisations of the machine. Furthermore, continuing our research with other groups of potential users and stakeholders and investigating relationships between postinteraction acceptance of the robot and functional capacity or needs of older persons might lead to new insights.

## Figures and Tables

**Table 1:** UNRAQ results before and after interaction with the robot.

Area	Statement	1st Assessment Mean ± SD (Median)	2nd Assessment Mean ± SD (Median)	Wilcoxon Pair Order Test (*p*)
A. INTERACTION WITH THE ROBOT AND TECHNICAL ISSUES	A1 The robot should be a companion of the elderly person	3.9 ± 1.4 (4)	4.4 ± 1.1 (4)	**<0.001**
	A2 The robot should be an assistant of the elderly person	4.1 ± 1.3 (5)	4.6 ± 0 (5)	**<0.005**
	A3 The robot should be a useful device of the elderly person (something to be used when needed, with no other interaction)	4.3 ± 1.2 (5)	4.7 ± 0.7 (5)	**<0.001**
	A4 The elderly are prepared to interact with a robot	1.8 ± 1.0 (1)	2.2 ± 1.4 (2)	**<0.005**
	A5 The elderly are able to manage with the robot	2.6 ± 1.3 (2)	2.7 ± 1.4 (3)	0.366270
	A6 The elderly want to increase their knowledge about the robots to be able to operate them	3.6 ± 1.4 (4)	3.8 ± 1.4 (4)	0.208229
	A7 The robot should instruct the elderly person what to do in case of a problem with its operation	4.4 ± 1.0 (5)	4.6 ± 1.0 (5)	0.082072
	A8 The robot should be customisable (adjusted to individual user preferences and needs)	4.3 ± 1.1 (5)	4.7 ± 0.9 (5)	**<0.001**
	A9 The elderly should be able to choose the functions of the robot they want to use and disable other ones	4.3 ± 1.2 (5)	4.5 ± 1.0 (5)	0.071043
	A10 If the robot has been switched off by the owner, it should reactivate automatically (after a specific period) so that it is not forgotten in off mode	4.3 ± 1.2 (5)	4.5 ± 1.1 (5)	0.236306
B. ASSISTIVE ROLE OF THE ROBOT	B1 The robot should increase the safety of the elderly home, e.g., locking doors, detecting leaking gas etc.	4.7 ± 0.8 (5)	4.8 ± 0.7 (5)	0.365517
	B2 The robot should help the elderly to preserve their memory function, e.g., by playing memory games with them	4.6 ± 0.9 (5)	4.7 ± 0.8 (5)	0.097018
	B3 The robot should encourage and guide the elderly to perform physical exercises	4.5 ± 1.1 (5)	4.6 ± 0.9 (5)	0.162042
	B4 The robot should provide advice about a healthy diet	4.1 ± 1.3 (5)	4.3 ± 1.1 (5)	0.092461
	B5 The robot should monitor the environment (temperature, humidity) and suggest air conditioning adjustment or windows opening	4.5 ± 1.0 (5)	4.6 ± 0.9 (5)	0.130593
	B6 The robot should measure physiological parameters (blood pressure, heart rate, body temperature) of the elderly person	4.7 ± 0.9 (5)	4.6 ± 0.9 (5)	0.600458
	B7 The robot should monitor the amount of food and fluid intake of the owner	3.9 ± 1.4 (5)	4.1 ± 1.4 (5)	0.288922
	B8 The robot should remind the elderly about appointments	4.5 ± 1.0 (5)	4.6 ± 1.0 (5)	0.330880
	B9 The robot should remind the elderly about medication	4.6 ± 0.9 (5)	4.8 ± 0.8 (5)	0.178957
	B10 The robot should remind about meals times, drinks	4.2 ± 1.3 (5)	4.4 ± 1.2 (5)	0.127508
	B11 The robot should observe the behaviour of the elderly person to detect falls or changes due to illness	4.7 ± 0.8 (5)	4.8 ± 0.6 (5)	0.186572
	B12 The robot should call the centre in case of emergency	4.9 ± 0.5 (5)	4.8 ± 0.7 (5)	0.444587
	B13 The robot should help the owner to find lost objects (e.g., glasses, keys)	4.6 ± 0.9 (5)	4.6 ± 0.9 (5)	0.061287
C. SOCIAL ASPECTS	C1 The robot could decrease the sense of loneliness and improve the mood of the elderly person	3.8 ± 1.4 (4)	4.3 ± 1.1 (4)	**<0.0005**
	C2 The robot could encourage the elderly to enhance their contacts with friends	4.2 ± 1.1 (5)	4.4±1.1 (5)	0.104077
	C3 The robot should initiate contacts with others (calling friends, initiating skype conversations)	4.2 ± 1.3 (5)	4.3 ± 1.2 (5)	0.262570
	C4 The robot should have entertainment functions (e.g., gaming partner, reading aloud or playing music function)	4.5 ± 1.0 (5)	4.6 ± 1.0 (5)	0.270767
	C5 The robot should detect the owner’s mood (facial expression)	4.2 ± 1.2 (5)	4.3 ± 1.2 (5)	0.458659
	C6 The robot should accompany the owner in everyday activities (watching TV, preparing meals)	4.0 ± 1.3 (5)	4.1 ± 1.3 (5)	0.883143
D. ETHICAL ISSUES	D1 The elderly person should have control over the robot	4.2 ± 1.2 (5)	4.3 ± 1.2 (5)	0.616456
	D2 The elderly person should be able to send the robot to its place/docking station and keep it there	4.2±1.0 (5)	4.4 ± 1.1 (5)	0.272291
	D3 It is acceptable that the robot informs a family member or caregiver about the older person’s behaviour/health problems	4.2 ± 1.0 (5)	4.6±1.0 (5)	**<0.005**
	D4 The elderly person should be able to switch off the robot in specific situations (friends’ visits, privacy reasons etc.)	4.5 ± 0.9 (5)	4.6 ± 0.9 (5)	0.336526
	D5 It is acceptable that the robot will have much information about the user (social, medical, others)	4.1 ± 1.3 (5)	4.3 ± 1.1 (5)	0.060194

## Data Availability

The data presented in this study are available from the corresponding author upon reasonable request.

## Supplementary Materials

The following supporting information can be downloaded at: https://www.mdpi.com/article/10.3390/s22051717/s1, Table S1: The determinants of participants’ opinion changes: percentages of participants whose opinions worsened after interaction with the robot; Table S2: Percentages of participants whose opinions worsened after interaction with the robot in relation to the scores of Mini Mental State Examinstion (MMSE) and Barthel Index.
