# Supplementary material for: Robots in Eldercare: How Does a Real-World Interaction with the Machine Influence the Perceptions of Older People?
Source: Sensors (Basel). 2022 Feb 22;22(5):1717. doi: 10.3390/s22051717 (PMC8915103; doi:10.3390/s22051717)
Supplement: Supplementary file 1 [file sensors-22-01717-s001.zip › sensors-1545385-supplementary.pdf]

Table S1. The determinants of participants' opinion changes: percentages of participants whose opinions worsened after interaction with the robot

Area of the questionnaire	Age: <80 years vs. ≥80 years	Gender: females vs. males	Education: primary and below vs. above primary	Ease of use of technolo–gical devices: 1-3 vs. 4-5*	Self-perception of health: 1-3 vs. 4-5*	Self-perception of fitness: 1-3 vs. 4-5*
**A: INTERACTION WITH THE ROBOT AND TECHNICAL ISSUES**						
A1 The robot should be a companion of the elderly person	9.2% vs. 16.7%	17.2% vs. 6.1%	10.6% vs. 13.6%	9.5% vs. 16.3%	11.8% vs. 13.5%	14.1% vs. 9.5%
A2 The robot should be an assistant of the elderly person	4.6% vs. 16.7%	12.5% vs. 6.1%	10.6% vs. 9.1%	11.1% vs. 8.2%	9.2% vs. 10.8%	11.3% vs. 7.1%
A3 The robot should be a useful device of the elderly person (something to be used when needed, with no other interaction)	7.7% vs. 6.3%	7.8% vs. 6.1%	4.3% vs 9.1%	3.2% vs. 12.2%	10.5% vs. 0.0%	7.0% vs. 7.1%
A4 The elderly are prepared to interact with a robot	20.0% vs. 16.7%	14.1% vs. 24.5%	12.8% vs. 22.7%	14.3% vs. 24.5%	19.7% vs. 16.2%	16.9% vs. 21.4%
A5 The elderly are able to manage with the robot	29.2% vs. 31.3%	26.6% vs. 34.7%	27.7% vs. 31.8	25.4% vs. 36.7%	30.3% vs. 29.7%	29.6% vs. 31.0%
A6 The elderly want to increase their knowledge about the robots to be able to operate them	26.2% vs. 20.8%	23.4% vs. 24.5%	19.2% vs. 27.3%	17.5% vs. 32.7%	23.7% vs. 24.3%	29.6% vs. 14.3%
A7 The robot should instruct the elderly person what to do in case of problems with its operation	9.2% vs. 16.7%	10.9% vs. 14.3%	10.6% vs. 13.6%	7.9% vs. 16.3%	10.5% vs. 16.2%	9.9% vs. 16.7%
A8 The robot should be customizable (adjusted to individual user preferences and needs)	6.2% vs. 6.3%	7.8% vs. 4.1%	4.3% vs. 7.6%	6.4% vs. 6.1%	6.6% vs. 5.4%	4.2% vs. 9.5%
A9 The elderly should be able to choose the functions of the robot they want to use and disable other ones	18.5% vs. 8.3%	10.9% vs. 18.4%	12.8% vs. 15.2%	15.9% vs. 12.2%	18.4% vs. 5.4%	18.3% vs. 7.1%
A10 If the robot has been switched off by the owner, it should reactivate automatically (after a specific period) so that it is not forgotten in off mode	13.9% vs. 12.5%	15.6vs. 10.2%	6.4% vs. 18.2%	14.3% vs. 12.2%	13.2% vs. 13.5%	12.7% vs. 14.3%
**B: ASSISTIVE ROLE OF THE ROBOT**						
B1 The robot should increase the safety of the elderly home: e.g. locking doors, detecting leaking gas etc.	3.1% vs. 6.3%	6.3% vs. 2.0%	6.4% vs. 3.0%	3.2% vs. 6.1%	4.0% vs. 5.4%	4.2% vs. 4.8%
B2 The robot should help the elderly to preserve their memory function, e.g. by playing memory games with them	**6.2% vs. 18.8%** $p<0.05$	14.1% vs. 8.2%	4.3% vs. 16.7%	16.7% vs. 14.3%	11.8% vs. 10.8%	9.9% vs. 14.3%
B3 The robot should encourage and guide the elderly to perform physical exercises	10.8% vs. 14.6%	14.1% vs. 10.2%	4.3% vs. 16.7%	16.7% vs. 12.2%	14.5% vs. 8.1%	12.7% vs. 11.9%
B4 The robot should provide advice about a healthy diet	15.4% vs. 16.7%	15.6% vs. 16.3%	17.0% vs. 15.2%	**22.2% vs. 8.2%** $p<0.05$	18.4% vs. 10.8%	14.1% vs. 19.1%
B5 The robot should monitor the environment (temperature. humidity) and suggest air conditioning adjustment or windows opening	6.2% vs. 12.5%	10.9% vs. 6.1%	8.5% vs. 9.1%	9.1% vs. 8.2%	10.5% vs. 5.4%	12.7% vs. 2.4%
B6 The robot should measure physiological parameters (blood pressure. heart rate. body temperature) of the elderly person	16.9% vs. 10.4%	9.4% vs. 20.4%	14.9% vs. 13.6%	13.6% vs. 10.2%	14.5% vs. 13.5%	14.1% vs. 14.3%
B7 The robot should monitor the amount of food and fluid intake of the owner	20.0% vs. 22.9%	18.8% vs. 24.5%	23.4% vs. 19.7%	19.7% vs. 18.4%	23.7% vs. 16.2%	21.1% vs. 21.4%
B8 The robot should remind the elderly about appointments	12.3% vs. 8.3%	7.8% vs. 14.3%	17.0% vs. 6.1%	6.4% vs. 16.3%	13.2% vs. 5.4%	8.5% vs. 14.3%
B9 The robot should remind the elderly about medication	7.7% vs. 4.2%	3.1% vs. 10.2%	6.4% vs. 6.1%	6.1% vs. 12.2%	6.6% vs. 5.4%	7.0% vs. 4.8%
B10 The robot should remind about meals times. drinks	20.0% vs. 16.7%	18.8% vs. 18.4%	10.6% vs. 24.2%	24.2% vs. 22.5%	18.4% vs. 18.9%	18.3% vs. 19.1%

B11 The robot should observe the behaviour of the elderly person to detect falls or changes due to illness	7.7% vs. 6.3%	7.8% vs. 6.1%	10.6% vs. 4.6%	4.6% vs. 6.1%	7.9% vs. 5.4%	5.6% vs. 9.5%
B12 The robot should call the centre in case of emergency	7.7% vs. 2.1%	1.6% vs. 10.2%	6.4% vs. 4.6%	4.6% vs. 8.2%	6.6% vs. 2.7%	7.0% vs. 2.4%
B13 The robot should help the owner to find lost objects (e.g. glasses, keys)	4.6% vs. 4.2%	7.8% vs. 0.0%	2.1% vs. 6.1%	3.2% vs. 6.1%	5.3% vs. 2.7%	4.2% vs. 4.8%
**C: SOCIAL ASPECTS OF USING THE ROBOT**						
C1 The robot could decrease the sense of loneliness and improve the mood of the elderly person	10.8% vs. 16.7%	15.6% vs. 10.2%	8.5% vs. 16.7%	9.5% vs. 18.4%	14.5% vs. 10.8%	15.5% vs. 9.5%
C2 The robot could encourage the elderly to enhance their contacts with friends	7.7% vs. 18.8%	14.1% vs. 10.2%	6.4% vs. 16.7%	11.1% vs. 14.3%	11.8% vs. 13.5%	12.7% vs. 11.9%
C3 The robot should initiate contacts with others (calling friends, initiating skype conversations)	13.9% vs. 18.8%	18.8% vs. 12.2%	10.6% vs. 19.7%	14.3% vs. 18.4%	17.1% vs. 13.5%	18.3% vs. 11.9%
C4 The robot should have entertainment functions (e.g. gaming partner, reading aloud or playing music function)	10.8% vs. 14.6%	12.5% vs. 12.2%	10.6% vs. 13.6%	9.5% vs. 16.3%	14.5% vs. 8.1%	14.1% vs. 9.5%
C5 The robot should detect the owner's mood (facial expression)	20.0% vs. 14.6%	18.8% vs. 16.3%	19.2% vs. 16.7%	17.5% vs. 18.4%	22.4% vs. 8.1%	19.7% vs. 14.3%
C6 The robot should accompany the owner in everyday activities (watching TV, preparing meals)	23.1% vs. 25.0%	29.7% vs. 16.3%	21.3% vs. 25.8%	27.0% vs. 20.4%	26.3% vs. 18.9%	23.9% vs. 23.8%
**D: ETHICAL ISSUES**						
D1 The elderly person should have control over the robot	18.5% vs. 20.8%	18.8% vs. 20.4%	17.0% vs. 21.2%	17.5% vs. 22.5%	23.7% vs. 10.8%	19.7% vs. 19.1%
D2 The elderly person should be able to send the robot to its place/docking station and keep it staying there	21.5% vs. 12.5%	12.5% vs. 24.5%	21.3% vs. 15.2%	19.1% vs. 16.3%	17.1% vs. 18.9%	18.3% vs. 16.7%
D3 It is acceptable that the robot informs a family member or caregiver about the older person's behaviour/health problems	7.7% vs. 8.3%	6.3% vs. 10.2%	6.4% vs. 9.1%	9.5% vs. 6.1%	10.5% vs. 2.7%	9.9% vs. 4.8%
D4 The elderly person should be able to switch off the robot in specific situations (friends' visits, privacy reasons etc.)	13.9% vs. 16.7%	10.9% vs. 20.4%	12.8% vs. 16.7%	14.3% vs. 16.3%	14.5% vs. 16.2%	16.9% vs. 11.9%
D5 It is acceptable that the robot will have much information about the user (social, medical, others)	18.5% vs. 8.3%	**7.8% vs. 22.5%** $p<0.05$	8.5% vs. 18.2%	14.3% vs. 14.3%	18.4% vs. 5.4%	12.7% vs. 16.7%

* *Ease of use of technological devices*, *Self-perception of health* and *Self-perception of fitness* were rated on a 5-point scale: from 1 (worst) via 3 (neutral) to 5 (best).

Table S2. Percentages of participants whose opinions worsened after interaction with the robot in relation to the scores of Mini Mental State Examinstion (MMSE) and Barthel Index

Area of the questionnaire	MMSE: 15-19 points vs. ≥20points	Barthel Index: <80 points vs. ≥85 points
**A: INTERACTION WITH THE ROBOT AND TECHNICAL ISSUES**		
A1 The robot should be a companion of the elderly person	18.2% vs. 10.3%	13.3% vs. 10.7%
A2 The robot should be an assistant of the elderly person	18.2% vs. 7.7%	8.9% vs. 10.7%
A3 The robot should be a useful device of the elderly person (something to be used when needed, with no other interaction)	4.6% vs. 7.7%	2.2% vs. 10.7%
A4 The elderly are prepared to interact with a robot	18.2% vs. 18.0%	17.8% vs. 17.9%
A5 The elderly are able to manage with the robot	40.9% vs. 25.6%	35.6% vs. 25.0%
A6 The elderly want to increase their knowledge about the robots to be able to operate them	22.7%vs. 24.4%	24.4% vs. 23.2%
A7 The robot should instruct the elderly person what to do in case of problems with its operation	0.0% vs. 14.1%	11.1% vs. 12.5%
A8 The robot should be customizable (adjusted to individual user preferences and needs)	4.6% vs. 6.4%	6.7% vs. 5.4%
A9 The elderly should be able to choose the functions of the robot they want to use and disable other ones	0.0% vs. 14.1%	13.3% vs. 8.9%
A10 If the robot has been switched off by the owner, it should reactivate automatically (after a specific period) so that it is not forgotten in off mode	4.6% vs. 16.7%	13.3% vs. 14.3%
**B: ASSISTIVE ROLE OF THE ROBOT**		
B1 The robot should increase the safety of the elderly home: e.g. locking doors, detecting leaking gas etc.	4.6% vs. 3.9%	4.4% vs. 3.6%
B2 The robot should help the elderly to preserve their memory function, e.g. by playing memory games with them	13.6% vs. 9.0%	**2.2% vs. 16.1%** *p<0.05*
B3 The robot should encourage and guide the elderly to perform physical exercises	18.2% vs. 9.0%	6.7% vs. 16.1%
B4 The robot should provide advice about a healthy diet	18.2% vs. 15.4%	8.9% vs. 21.4%
B5 The robot should monitor the environment (temperature. humidity) and suggest air conditioning adjustment or windows opening	13.6% vs. 6.4%	4.4% vs. 10.7%
B6 The robot should measure physiological parameters (blood pressure. heart rate. body temperature) of the elderly person	18.2% vs. 14.1%	13.3% vs. 16.1%
B7 The robot should monitor the amount of food and fluid intake of the owner	22.7% vs. 23.1%	20.0% vs. 25.0%
B8 The robot should remind the elderly about appointments	18.2% vs. 10.3%	8.9% vs. 14.3%
B9 The robot should remind the elderly about medication	4.6% vs. 7.7%	4.4% vs. 8.9%
B10 The robot should remind about meals times. drinks	22.7% vs. 19.2%	15.6% vs. 23.2%
B11 The robot should observe the behaviour of the elderly person to detect falls or changes due to illness	13.6% vs. 5.1%	8.9% vs. 5.4%
B12 The robot should call the centre in case of emergency	4.6% vs. 5.1%	6.7% vs. 3.6%
B13 The robot should help the owner to find lost objects (e.g. glasses, keys)	0.0% vs. 5.1%	4.4% vs. 3.6%

C: SOCIAL ASPECTS OF USING THE ROBOT		
C1 The robot could decrease the sense of loneliness and improve the mood of the elderly person	13.6% vs. 11.5%	11.1% vs. 12.5%
C2 The robot could encourage the elderly to enhance their contacts with friends	13.6% vs. 12.8%	11.1% vs. 16.1%
C3 The robot should initiate contacts with others (calling friends, initiating skype conversations)	18.2% vs. 14.1%	11.1% vs. 17.9%
C4 The robot should have entertainment functions (e.g. gaming partner, reading aloud or playing music function)	13.6% vs. 11.5%	8.9% vs. 14.3%
C5 The robot should detect the owner's mood (facial expression)	18.2% vs. 18.0%	17.8% vs. 17.9%
C6 The robot should accompany the owner in everyday activities (watching TV, preparing meals)	9.1% vs. 25.6%	17.8% vs. 25.0%
**D: ETHICAL ISSUES**		
D1 The elderly person should have control over the robot	22.7% vs. 14.1%	17.8% vs. 16.1%
D2 The elderly person should be able to send the robot to its place/docking station and keep it staying there	27.3% vs. 14.1%	13.3% vs. 19.6%
D3 It is acceptable that the robot informs a family member or caregiver about the older person's behaviour/health problems	4.6% vs. 9.0%	15.6% vs. 3.6%
D4 The elderly person should be able to switch off the robot in specific situations (friends' visits, privacy reasons etc.)	22.7% vs. 12.8%	15.6% vs. 14.3%
D5 It is acceptable that the robot will have much information about the user (social, medical, others)	18.2% vs. 14.1%	11.1% vs. 19.6%
